# Capacity building and community of practice for women community health workers in low-resource settings: long-term evaluation of the Mobile University For Health (MUH)

**DOI:** 10.3389/fgwh.2024.1304954

**Published:** 2024-05-20

**Authors:** Hady Naal, Reem Alaeddine, Dayana Brome, Tracy Daou, Laura Hudroj, Israa el Sayed, Racha Soubra, Joanne Hokayem, Mohamad Ghalayini, Waed Slim, Shadi Saleh

**Affiliations:** ^1^ Global Health Institute at the American University of Beirut, Beirut, Lebanon; ^2^ Department of Health Management, Evaluation and Policy, University of Montreal, Montreal, QC, Canada; ^3^ School of Public Health, Harvard University, Cambridge, MA, United States; ^4^ Faculty of Health Sciences, American University of Beirut, Beirut, Lebanon

**Keywords:** refugee, health, capacity building, evaluation, Lebanon, community health worker, task-shifting

## Abstract

**Background:**

Lebanon has been facing a series of crises, significantly increasing health challenges, and straining its healthcare infrastructure. This caused deficiencies in the system's ability to attend to population health needs, and it profoundly impacted vulnerable and refugee communities who face additional challenges accessing healthcare services. In response, the Global Health Institute at the American University of Beirut designed and implemented the Mobile University for Health (MUH), which promotes task-shifting through capacity building complemented by communities of practice (CoP). The program aimed to prepare vulnerable women to assume the role of community health workers (CHW) within their communities, and to promote positive health knowledge and behaviours.

**Methods:**

A mixed-methods approach was used to evaluate MUHs' three certificates (women's health, mental health and psychosocial support, and non-communicable diseases). Implementation took place between 2019 and 2022, with 83 CHWs graduating from the program. Short-term data including knowledge assessments, course evaluations, and community member feedback surveys were collected. 93 semi-structured interviews with CHWs and 14 focus group discussions with community members were conducted to evaluate the long-term impact of the capacity building and CoP components.

**Results:**

Data revealed multiple strengths of the initiative, including increased access to education for the community, effectiveness of blended learning modality, successful planning and delivery of CoP sessions, and improved knowledge, skills, and health behaviours over time. The supplementary CoP sessions fostered trust in CHWs, increased community empowerment, and increased leadership skills among CHWs. However, some challenges persisted, including limited access to healthcare services, implementation logistical issues, difficulties with some aspects of the learning modality, and some resistance within the communities.

**Conclusion:**

MUH promoted and improved positive health knowledge and behaviours within targeted vulnerable populations in Lebanon. The supplementary CoP component proved instrumental in empowering CHWs and enhancing their impact within their communities. The study highlights the need for ongoing training and support for CHWs and underscores the importance of continued investment and adaptation of such initiatives through a gendered lens. This evaluation provides evidence on the successes of a capacity building model that has strong potential for scale and replication across health topics in conflict-affected contexts.

## 1 Introduction

### 1.1 Context

Conflicts and displacement have been associated with increases in global health concerns affecting people's mental and physical health (1). In the Middle East and North Africa (MENA) region, health systems tend to face substantial challenges, compounded by the effects of multiple endemic conflicts and crises, along with an influx of refugees in the area. Most recently, after the breakout of the war in Syria in 2011, around 12 million Syrians fled the country and have been displaced in neighbouring countries (2).

Lebanon, a country that is already grappling with social, political, and economic turmoil, hosts over 1.5 million refugees (3). This in turn stretched its already strained healthcare system, affecting the availability of resources, provision of services, and operation of healthcare staff (4, 5). Lebanon also suffered from multiple simultaneous crises since 2019 that have compromised the response of its already unstable healthcare system, exacerbating its capacity to attend to population health needs (6, 7). For instance, the economic crisis Lebanon faced is arguably one of the worst globally in recent history whereby the Lebanese pound lost over 80% of its value relative to the US dollar. This crisis not only burdened the healthcare system financially but also triggered a massive exodus of trained professional health workers seeking better income generating opportunities abroad (8, 9). By 2021, almost 40% of skilled medical doctors and 30% of registered nurses had fled the country for better opportunities (10). On August 4th, 2020, an explosion hit Beirut's port, causing hundreds of deaths and thousands of injuries, as well as significant internal displacement (11). According to the World Health Organization (12) the blast damaged over half of the health infrastructure in the area, destroying facilities, and affecting hospitalization. Furthermore, at the time, the spread of COVID-19 caused additional stress in the system, aggravating the struggle to access health services (7).

In this context, although the general population was severely impacted by the above, refugees and other vulnerable groups in particular were especially affected. For example, seeking healthcare services became even more difficult for them, primarily due to financial and logistical factors (13–15). Beyond those, refugees in Lebanon usually fear mistreatment and discrimination when seeking medical attention (16) as large numbers of Syrians in Lebanon report worse treatment than others because of their nationality (17). Syrians form the predominant portion of Lebanon's refugee population and over 25% of the general population, contributing significantly to the demographic facing these challenges (18). Another prominent issue is their lack of awareness on health-related matters as a result of low health literacy (19, 20). Studies underline the limited knowledge of risk factors, symptoms, and available healthcare services for mental and physical issues and knowledge of available services (21, 22). This is particularly pronounced among refugees (23, 24). Health literacy has been associated with poor health status, inadequate knowledge on prevention measures, and an increase in chronic diseases, intensifying the burden on the healthcare system (25).

Therefore, addressing gaps in access to healthcare services and mitigating health risks, requires a concerted effort to enhance health literacy, improve preventive measures, promote positive health behaviours, and facilitate the dissemination of information regarding available services and referrals (15, 26, 27).

### 1.2 Task-shifting to bridge the health gap

Task-shifting, a strategy endorsed by the World Health Organization, involves redistributing specific healthcare tasks from highly trained professionals to individuals within the community who have varying degrees of training or education (28). This strategy is particularly useful in resource-constrained settings such as Lebanon where shortages of skilled workers pose a threat to healthcare system responses. Studies have shown that task-shifting can address health resource shortages in fragile settings (29). This has been established in relation to multiple health topics, by allocating certain tasks or roles to trained individuals from the community known as Community Health Workers (CHW), which can help with prevention, control, referral, and management of diseases, especially in remote areas where access to knowledge and health services is limited (30, 31). CHW training programs have been successful in addressing long-term gaps in health systems by delivery of preventative and curative services on various issues (32, 33).

One way to disseminate health information across communities is through Communities of Practice (CoP). CoPs can be formal or informal networks and gatherings where individuals from a given community convene to learn, discuss, and deepen their knowledge in certain topics (34). CoP initiatives have increased in recent years, and are being used to spread public health knowledge, improve connections between individuals and healthcare systems, and allow individuals to discuss sensitive issues in safe spaces in their communities (35–37). Thus, CoPs can be crucial facilitators of task-shifting, playing a pivotal role in enabling CHWs to become effective leaders within their communities and conveyors of health information. Despite the plethora of studies emphasizing the importance of CoP frameworks, there is little to no evidence of such interventions covering vulnerable populations in the MENA region. Given the challenges described so far in Lebanon, this approach can be beneficial in targeting several prominent health issues in vulnerable communities affecting refugees and local populations.

It has been previously shown that training CHWs to promote health and provide basic health services within their communities can be promising. However, comprehensive evaluations of community-level impact of these interventions remain limited (38, 39). For instance, a recent systematic review of global health capacity building initiatives in Low-and Middle-Income Countries (LMICs) in the MENA region found that despite their potential to engage with underserved populations and promote health education in resource-limited environments, CHWs are rarely targeted by these interventions (40). Another review highlighted the importance of implementing and adequately evaluating CHW training programs in the MENA region, to inform researchers and improve health outcomes in the area (41).

According to a recent systematic review assessing the health needs of Syrian refugees in the MENA region, women's health, mental health, and Non-Communicable Diseases (NCDs) were identified as the most prevalent and pressing health concerns in the community (2). The review highlighted the struggles that women, specifically Syrian refugees, have in accessing healthcare services and the fact that women's health challenges were highly prevalent among them specifically in relation to maternal health, birth complications, and poor reproductive health (42). The study highlighted the unaddressed needs of the refugee community and called for targeting refugees for reproductive health services as well as psychosocial support referral due to the elevated risk of violence against women in the community.

Mental health is yet another prevalent issue, which is also a global health concern and a cause for disability worldwide (26). This is especially pronounced in LMICs, whereby over 75% of individuals with mental illness remain untreated (26, 43). In Lebanon, it is estimated that one out of every four people in Lebanon suffers from some form of mental illness (44). Despite this, only a fifth of individuals with mental illness in Lebanon seek professional mental health care (45). The prevalence of mental health disorders among Syrian refugees in Lebanon is high, specifically Major Depressive Disorder (44%), and PTSD (27%), coupled with limited access to mental health services (46, 47). A more recent study estimated that one in four Syrian refugees meet the criteria for depression, and women are more likely to be affected by it (48). Factors such as limited mental health workforce, prevailing social stigma, and limited awareness and trust in the availability and the quality of the mental health care services are major barriers to accessing mental health care in Lebanon (26, 44).

Finally, NCDs, which accounted for 74% of deaths in the MENA region in 2018, are especially prevalent among Syrian refugees in Lebanon (49). A 2014 health access survey on the burden of NCDs among Syrian refugees found that half of the households sampled in the study reported having at least one member living with one or more NCDs (50). Furthermore, despite the prevalence of NCDs among Syrian refugees, there is limited evidence of studies focusing on their prevention among displaced populations in the MENA region, and thus there is a need to prioritize prevention efforts accordingly.

### 1.3 The present study

In response to these challenges, the Global Health Institute (GHI) at the American University of Beirut (AUB) piloted a Capacity Building (CB) initiative in 2020 in two under-served areas in Lebanon, providing 42 CHWs with training on women's health (41). The goal was to target displaced and refugee women, to support them in compensating for lost opportunities for health education due to conflicts, and to train them to become CHWs serving their communities. As such, the CB program which is the training component of MUH, aimed to equip them with the skills necessary to promote health and positive behavior, to provide basic services, and to conduct referrals when needed within their communities The evaluation of the pilot implementation found that despite successful implementation of the program and increases in knowledge among CHWs, there was a need to integrate more practical and hands-on components within the training and to increase opportunities for engagement and practice within their communities. This was reflected in the fact that the dissemination of knowledge was limited, reaching only families and friends of CHWs who are within their immediate circle, rather than the wider community.

To tackle this issue, a Community of Practice component was introduced into the program by GHI to address the limitations of the pilot implementation by promoting broader dissemination of information, providing CHWs with important leadership skills, improving their capacity and credibility to work further in this field in the service of their communities. The CoP component provided the CHWs with a space to deliver the knowledge they acquired through the CB component to their wider communities, facilitating the spread of this information.

In light of the above, the present study reports a long-term evaluation of the full MUH program with the added CoP modality at the individual and community levels.

## 2 Methods

### 2.1 MUH CB and CoP programs

The program tackles three priority health topics, namely Women's Health, Mental Health and Psychosocial Support (MHPSS), and Non-Communicable Diseases (NCDs) (See Supplementary Material for training topics). The CB initiative's main objective is to equip CHWs with comprehensive knowledge and essential skills that they can effectively use and disseminate within their communities. The program included the use of pre-recorded videos and learning materials accessed through a laptop by the learners while interacting with the teaching assistant on-site. Building on these from the initial pilot initiative, the updated program incorporated more interactive components like pamphlets, posters, educational games, and others to enhance engagement.

The additional CoP initiative was designed and developed to boost the program through amplifying the dissemination of health knowledge, strengthening community interactions, and fostering a sense of empowerment among CHWs and community members. In addition to equipping CHWs with vital knowledge through the CB component, the CoP component supplemented it by developing their leadership skills.

As part of CoP, CHWs who demonstrated understanding of key information from the MUH certificates were selected to deliver formal awareness sessions within their respective communities, strategically optimizing the reach of health information initially delivered through the CB component. CoP was developed under the guidance of the Curriculum Development Committee at GHI and was informed by the women who participated in the MUH program along with the previous MUH evaluation.

Planning for the MUH initiative was based on conversations and partnerships with local Non-Governmental Organizations (NGOs), municipalities, Primary Healthcare Centers (PHCs), and subject matter experts, along with the input of the Curriculum Development Committee. The planning phase allowed the development of tailored training sessions, recruitment of community members to become trained CHWs, reservation of suitable venues, and coordination with key members/stakeholders.

### 2.2 Project implementation

**Figure 1:**
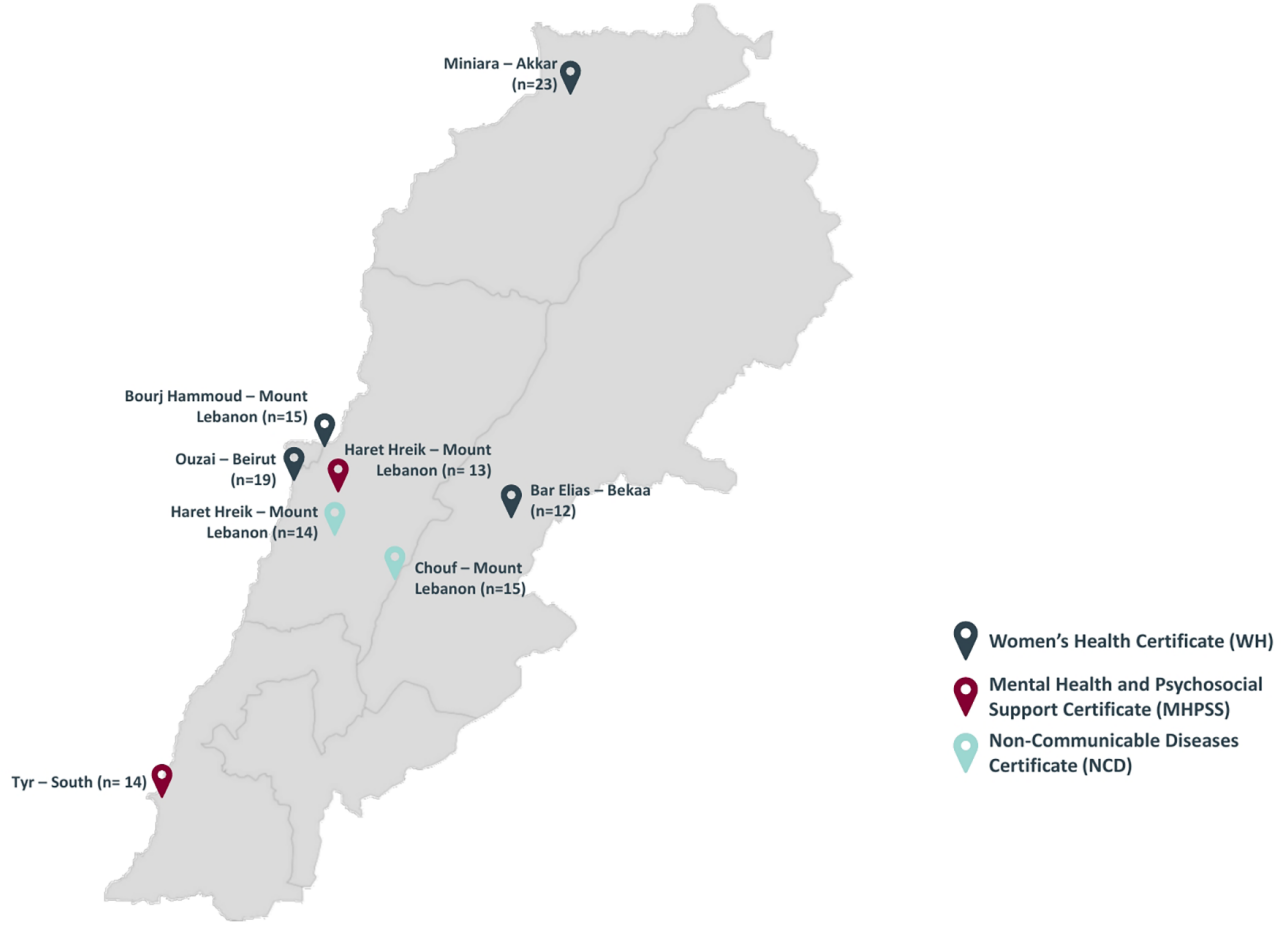
MUH capacity building implementation across Lebanon.

As part of its CB activities, the MUH team delivered 3 certificates between September 2020 and June 2022, to 6 different cohorts across several underserved regions in Lebanon (See Figure 1). Each certificate was delivered to 2 cohorts. A blended learning modality was employed, with different topics delivered across several areas as follows:

• Women's health (WH):
  ◦ Bar Elias (September–October 2020), *n* = 12
  ◦ Burj Hammoud (November–December 2020), *n* = 15
• Mental Health and Psychosocial Support (MHPSS):
  ◦ Sour (April–May 2021), *n* = 14
  ◦ Haret Hreik (February 2022), *n* = 13
• Non-Communicable Diseases (NCD):
  ◦ Haret Hreik (August–September 2021), *n* = 14
  ◦ Chouf (May–June 2022), *n* = 15

In total, 83 CHWs actively participated in the CB program (see Table 1 for demographics), with all courses and activities delivered in Arabic, the native language of the target population. The women were selected for the training based on meeting the following inclusion criteria:

a. Having official documentation of legal refugee status (if applicable).
b. Being between the ages of 18 and 55.
c. Having a minimum of middle school-level education.
d. Living in the area of implementation

**Table 1:** Demographics for MUH capacity building component.

	Women's health		MHPSS		NCDs	
	Bar Elias	Bourj Hammoud	Tyre	Haret Hreik	Haret Hreik	Chouf
Total	12	15	14	13	14	15
Nationality						
Lebanese	4 (33)	8 (53)	6 (43)	7 (54)	8 (57)	8 (53)
Syrian	7 (58)	7 (47)	8 (57)	6 (46)	4 (29)	7 (47)
Other	1 (8)	—	–	–	2 (14)	–
Age						
18–25	2 (17)	3 (20)	1 (7)	2 (15)	1 (7)	–
26–40	7 (58)	7 (58)	9 (64)	5 (39)	9 (64)	11 (73)
41–55	3 (25)	5 (33)	4 (29)	6 (46)	4 (29)	4 (27)
Education						
Below middle School	1 (8)	–	–	–	–	–
Middle school	3 (25)	2 (13)	–	3 (23)	–	3 (20)
High school	6 (50)	9 (60)	6 (43)	8 (62)	8 (57)	11 (73)
Vocational school	2 (17)	3 (20)	2 (14)	–	1 (7)	1 (7)
Bachelor's	–	1 (7)	2 (14)	2 (15)	3 (22)	–
Master's	–	–	2 (14)	–	2 (14)	–
Other	–	–	2 (14)	–	–	–

Data presented as *n* (%).

Although the initial criteria required a minimum of middle school-level education, one participant with education levels below middle school participated in the study. This decision was taken based on them being referred to the program and to ensure the availability of women participants. Prior to inclusion, an informal screening process was implemented in an interview format to ensure that they possessed the capacity to actively engage with and effectively articulate the content of the sessions.

The CoP component supplemented the CB program by selecting five women from each of the six cohorts, and the two cohorts in the pilot implementation. The selection process was based on (1) their attendance rate during the MUH program, (2) their performance on the knowledge assessment post completion of the MUH program, and (3) their interest in pursuing this opportunity. Should graduates be unable to take part in the CoP, the most suitable alternative was chosen from the remaining graduates, ensuring adherence to the eligibility criteria. A total of 40 CHWs from the CB were chosen for the CoP program, and 38 of them were able to lead the CoP sessions. A list was then subsequently delivered to the chosen trained CHW graduates who ranked health topics based on relevance in their communities. Based on the results of the ranking, a list of five pertinent health topics, each with several subtopics, was created for each region. To further support their leadership roles, each graduate was matched with a specific topic, accompanied by an information sheet comprising essential points to be addressed. The CHWs were compensated for their work preparing for and delivering the session, and community members received compensation for transportation.

**Figure 2:**
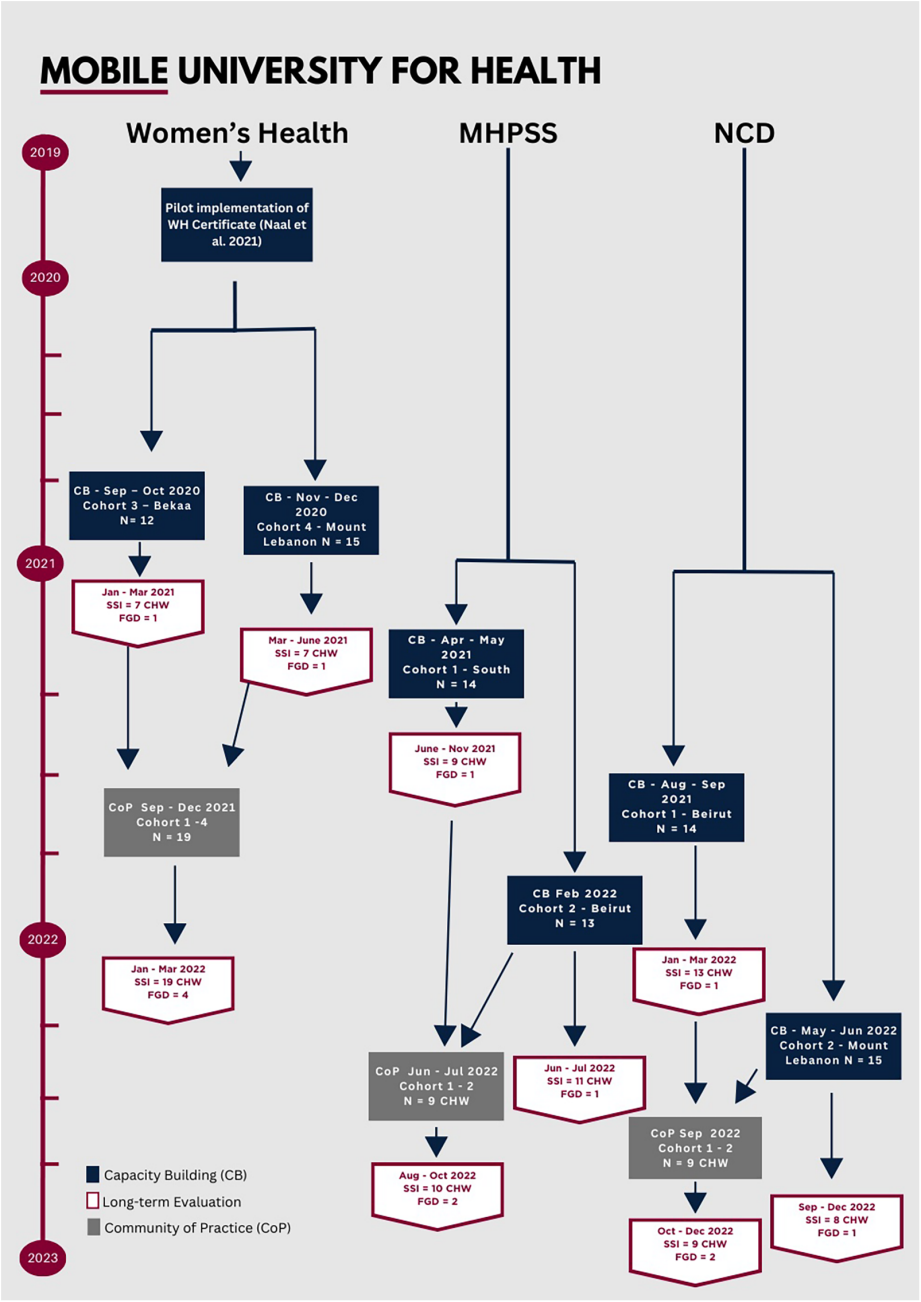
Timeline of implementation of the MUH initiative. CHW, community health worker; SSI, semi-structured interview; FGD, focus group discussion.

Each CHW graduate recruited up to ten women from their community who were not relatives. NGOs and municipality leaders supported in the recruitment of other women from the localities. A total of 243 women were reached through CoP sessions (see Table 2 for demographics). During the 3- to 4-h sessions, each trained CHW delivered their sections of allocated topics to community members. The sessions were delivered between September 2021 and September 2022 (See Figure 2).

**Table 2:** Demographics for MUH CoP component.

	Women's Health		MHPSS		NCDs	
All Cohorts	CHWs	Community Members	CHWs	Community Members	CHWs	Community Members
Total	20	119	9	38	9	54
Nationality						
Lebanese	9 (45)	63 (53)	4 (44)	24 (63)	5 (56)	34 (63)
Syrian	11 (55)	54 (45)	5 (56)	13 (34)	4 (44)	17 (31.5)
Other	—	2 (2)	–	1 (3)	–	2 (3.7)
Age						
18–25	3 (15)	26 (22)	1 (12)	11 (29)	–	8 (15.1)
26–40	13 (65)	37 (31)	4 (44)	9 (24)	6 (67)	22 (41.5)
41–55	4 (20)	33 (28)	4 (44)	13 (34)	3 (33)	16 (30.2)
56–80	—	23 (19)	–	5 (13)	–	7 (11.3)

Data presented as *n* (%).

### 2.3 Data collection tools and procedures

The data collection process included short and long-term individual and community-level data through a mixed-method approach (See Tables 3, 4). This approach combined qualitative and quantitative data from multiple sources to evaluate the impact of the MUH and CoP interventions. Informed by the Kirkpatrick model (51) for evaluating training programs our analysis focused on participants' reactions, learning outcomes, behavioural changes, and overall results. The study was approved by the Institutional Review Board (IRB) at the American University of Beirut (Approval number: SBS-2019-0403). Written consent was obtained from all participants before they took part in the CB and CoP sessions. The researchers who performed data collection and evaluation interviews were independent of the staff who developed, coordinated, or delivered the training. The following tools were used:

**Table 3:** Number of CHWs and evaluations for MUH's capacity building component.

CB cohorts	Learners	Knowledge assessment	Course evaluation/survey	Semi-structured interview	Focus group
Women's health cohort 3	12	12	12	7	1 (9)
Women's health cohort 4	15	15	15	7	1 (9)
MHPSS cohort 1	14	12	12	9	1 (6)
MHPSS cohort 2	13	13	13	11	1 (11)
NCD cohort 1	14	14	14	13	1 (9)
NCD cohort 2	15	15	15	8	1 (8)
Total	83	81	81	55	6

**Table 4:** Number of CHWs, community members, and evaluations for MUH's CoP component.

CoP cohorts	CHW's	Community members	Course evaluation/survey	Semi-structured interview	Focus group
COP women's health, 4 cohort	19	119	119	19	4 (36)
COP MHPSS, 2 cohorts	9	60	38	10	2 (17)
COP NCD, 2 cohorts	9	69	54	9	2 (20)
Total	37	248	211	38	8

#### 2.3.1 Short-term evaluation

##### 2.3.1.1 Knowledge assessment

To measure changes in participants' knowledge of the health topics before and after the MUH capacity building component, pre- and post-tests were administered in person to all CHWs who completed the training (*n *= 81). The tests were developed by the subject matter experts responsible for creating the MUH certificate content.

##### 2.3.1.2 Course evaluation survey

Self-reported surveys were administered to participants in-person at the end of the CB sessions to assess individual satisfaction with the certificate among the trainees (*n* = 81). The course evaluations included 16 quantitative questions rated on a 5-point Likert scale evaluating course delivery, content, and instructor performance, and (2) three open-ended questions addressing strengths and weaknesses of the courses along with recommendations for future training.

##### 2.3.1.3 Cop satisfaction survey

After participating in CoP sessions, community members completed the CoP satisfaction survey in-person (*n* = 211). This tool gathered data on general demographic information and asked 21 questions rated on a 5-point Likert scale addressing the content and activities within the sessions, the delivery process, and the performance of the CHWs graduates who led the sessions. Some participants were unable to complete the survey due to time constraints.

#### 2.3.2 Long-term evaluation

##### 2.3.2.1 Semi-structured interview

To assess the long-term impact of the CB sessions, semi-structured interviews (SSI) were conducted with CHWs via phone, lasting between 20 and 30 min (*n* = 55). These interviews followed a comprehensive guide, previously used in the pilot implementation of MUH (Refer to Supplementary Material) (41).

As for the CoP component, long-term impact was assessed through SSIs with questions pertaining specifically to the sessions (Refer to Supplementary Material), conducted via phone with the CHW graduates who led the CoP sessions (*n* = 38). These SSIs also lasted between 20 and 30 min and included nine questions that aimed to assess (1) the overall experience of the CHWs during the sessions, (2) the strengths and weaknesses of the sessions, and (3) their ability to perform their assigned activities and responsibilities.

All interviews were recorded with participant consent and transcribed verbatim in the original language.

##### 2.3.2.2 Focus group discussion

Another measure of long-term impact was addressed through Focus Group Discussions (FGDs) with community members after CB and CoP sessions (refer to Supplementary Material), lasting about 1 h each.

Six FGDs, one for every cohort, were conducted between 3 and 6 months post-delivery of certificate (*n* = 52). They included community members recruited from community centers, based on them having contact with trained CHWs. Community members were Syrian and Lebanese females.

Eight additional FGDs were also held with community members who took part in CoP sessions 3–6 months post completion (*n* = 73). FGD guides included eight questions addressing the activities presented, perceptions of the performance of the CHW graduates, and the effectiveness of the sessions in disseminating health information.

These FGDs were conducted in-person in a safe and private setting at a local community center.

### 2.4 Analysis

The quantitative data collected from knowledge assessments, course evaluations, and CoP surveys were managed and analysed using the Statistical Package for the Social Sciences (SPSS), with significance set at *p* < 0.05. The data from course evaluations and CoP surveys were reported using frequencies and percentages. For each course, mean scores for pre and post knowledge assessments were calculated, and paired *t*-tests were used to compare the differences between individual scores.

SSIs and FGDs were used to collect qualitative data, which were recorded, transcribed verbatim, and translated to English. Open coding was used to analyse all qualitative data, including open-ended questions from course evaluations. Each participant was given a unique code to protect their anonymity. The evaluation approach was informed by the Kirkpatrick model (52), and data analysis followed both a deductive and inductive approach, with themes informed by the previous MUH evaluation study, and new themes developed based on the new data. After open coding, preliminary codes were developed collaboratively by multiple researchers, and these codes were then grouped into broader categories based on their relationships and general themes. The authors met on a regular basis to discuss the research process and their experiences, and to enhance awareness of their own perspectives and biases to ensure a more transparent and nuanced interpretation of data.

## 3 Results

**Figure 3:**
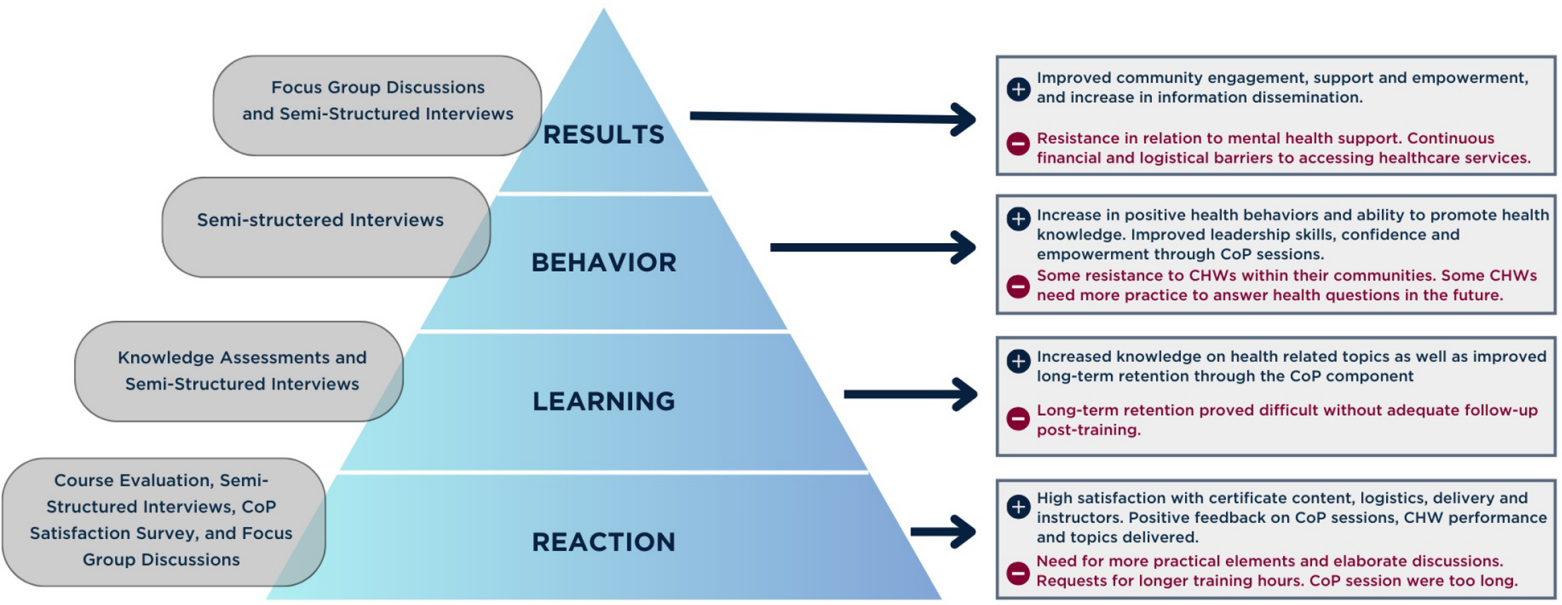
Evaluation and results through the Kirkpatrick model.

To evaluate the impact of the CB and CoP initiatives at the individual and community levels, findings from both short-term quantitative assessments and long-term qualitative insights are presented (See Figure 3 for general findings). The quantitative analysis showcases the findings from the knowledge assessments, course evaluations for the CB component, and CoP satisfaction surveys administered to community members (See Table 5, Supplementary Material). Data from SSIs with CHWs and FGDs with community members were combined into one analysis and triangulated. Findings were grouped into different emerging themes under two categories of strengths and weaknesses (see Supplementary Material).

**Table 5:** CHWs pre- and post- knowledge assessments.

Health topic	Cohort area	Pre-test		Post-test		Paired *t*-test		
		M	SD	M	SD	t	df	Sig.
Women's health	Bar Elias	64.08	6.36	81.5	10.21	−6.036	11	<0.001*
	Bourj Hammoud	61.73	14.77	75.87	12.51	−5.712	14	<0.001*
Mental health and psychosocial support	Tyre	63.87	9.09	77.61	13.44	−5.772	12	<0.001*
	Haret Hreik	52.94	11.99	67.51	14.09	−3.822	14	<0.001*
Non-communicable diseases	Haret Hreik	74.06	7.15	85.45	7.19	−8.189	13	<0.001*
	Chouf	61.79	11.99	80.47	10.92	−10.663	14	<0.001*

The number marked by an asterisk in the table points towards statistical significance (<0.05).

### 3.1 Strengths

Strengths of the CB and CoP sessions were grouped into seven themes: (1) access to education, (2) learning modality, (3) CoP logistics and delivery (4) knowledge acquisition & skillsets, (5) health-related behaviors, (6) individual and community empowerment, 8) significance of the program.

#### 3.1.1 Access to education

As seen in the findings of the course evaluation, CHWs found the content of the trainings relevant to their needs and reported in the interviews that the information they received was new and addressed the health challenges in their communities. Many expressed that this was their first time attending structured education in this type of setting and on the topics provided. The program offered women an opportunity to expand their knowledge in, and increase their exposure to, health-related education. CHWs explained that receiving information through the different employed modalities such as videos, PDFs, visuals, and others provided a new learning experience that they did not previously have access to.

*WH, CoP, FGD, CM.* “*[…] we did not know anything [before the activity] and now, little by little, we are becoming knowledgeable about diabetes and cancer and such things. Through this activity, all of us started to benefit more.*”

Specifically, the CoP component was perceived by CHWs as an aspect that strengthened their knowledge acquisition as well as a way for them to disseminate this information to community members in ways that they would not have previously been able to do. The CoP sessions were reported to be accessible to women from different socio-economic and educational backgrounds and locations, ensuring a wider scope for information dissemination.

*NCD, CoP, SSI, CHW.* “*The experience was nice because the women who came, there were things they haven't heard of and don't know about, even we learned from our colleagues*’ *things we did not know.*”

During the FGDs following the CoP sessions, community members emphasized their need for similar initiatives targeting the most prominent health issues and their interest in participating in them. This is consistent with high percentage of community members expressing their willingness to attend similar events in the future during the CoP satisfaction survey. They also expressed the need for more CHWs to further increase access to health information in the community. While the data from the surveys revealed that interacting and discussing with CHWs promoted learning and knowledge during the sessions, long-term evaluation through the FGDs showed that staying connected with the CHWs after the CoP awareness activities, according to some community members, fostered continued learning through the exchange of knowledge and ideas beyond formal events.

*NCD, CoP, FGD, CM.* “*Yes, a suggestion, if sessions like this follow-up on these topics, health-topics, are very very important to us and to our children*”*.*

#### 3.1.2 Learning modality

Several CHWs reported that the blended learning modality through which pre-recorded videos, visuals, and printouts were used, helped them understand the information efficiently. CHWs shared that the use of laptops was helpful when it came to understanding the information and following up with the instructors' explanations. It also helped them develop tech skills that they did not previously have, which they described as helpful to further pursue other educational opportunities independently.

Similarly, in-person interactions and explanations with instructors enhanced learners' engagement and their need to ask questions and receive feedback. The face-to-face modality was also perceived as particularly relevant in the MHPSS initiative as participants were able to observe the body language of the instructor when interacting and communicating with others with regard to mental health topics, and to be able to model the behaviour.

*MHPSS, CB, SSI, CHW.* “*[…] The instructor was clarifying how we should hold eye contact with the person we are talking to […] the interaction that was happening (through instructor and videos) was helping deliver the idea that the body language when talking to someone or teaching someone or listening to someone is very important.*”

A preference and advantage of the in-person component of the initiative was communicated as it facilitated discussions among CHWs and community members in a dedicated space, providing an opportunity for conversations on topics they would not usually have in their day to day lives. This allowed them to share various perspectives and ideas, and exposed them to real-life examples from within their communities. Another strength of the delivery method was the use of anatomical models to explore and explain the bodily functions and systems specifically pertaining to women's health and NCDs, as the visual component helped with absorption of this new information.

*WH, CoP, FGD, CM.* “*Everything was well-organized, the slides, the posters; all helped me to understand the content better. Especially the doctor was very helpful in explaining any medical information*”*.*

The CoP sessions further confirmed the effectiveness of utilizing a variety of platforms for information dissemination. CHWs found that these platforms assisted in delivery of information, and community members found that they kept them engaged and enhanced the CHWs capabilities when presenting during the sessions. The incorporation of lectures, posters, and activities as part of the sessions proved to be instrumental in simplifying the learning process and comprehending the concepts covered in them. This approach significantly contributed to the community members ability to grasp and retain the information presented.

#### 3.1.3 Cop logistics and delivery

According to CHWs, having the sessions planned and prepared in advance equipped them with the confidence needed to deliver the sessions successfully. The sessions encouraged increased participation and interaction between community members and CHWs tackling health topics that are relevant to the community's needs. Another point that was highlighted in the interviews is that individuals with lower educational levels also had access to the CoP sessions and were able to actively enrich the discussions and present insightful questions.

##### 3.1.3.1 Performance of CHWs during CoP sessions

Community members who attended CoP sessions provided positive feedback on the willingness of CHWs and doctors to address their inquiries, and to engage in discussions. Also, CHWs were perceived as confident and knowledgeable when leading the sessions, they were eager to promote positive health knowledge and behaviours. Another notable aspect was the CHWs' ability to create a safe environment were members felt comfortable sharing their perspectives, stories, and sensitive experiences.

*MHPSS, CoP, FGD, CM.* “*Aside from the information that you were giving us that was useful, the social engagement, you find that everyone is around you, that in itself is therapeutic*”*.*

##### 3.1.3.2 Trust in CHWs and MUH initiative

Trust placed in CHWs is a particularly important aspect of MUH, and the feedback provided by members in both the CoP surveys and the FGDs suggested that this was established during the sessions. Most community members said they sought help and guidance from CHWs after they earned a certificate and delivered the CoP sessions. The information communicated during the sessions was considered legitimate and trustworthy as the source is a recognized and established organization. Community members' real-life experiences aligned with the information communicated by the CHWs, fostering more trust due to their relatability. Furthermore, several community members reported that the CHWs' development and positive transformation, such as improved ties with family members and the adoption of a positive outlook and way of life, contributed to their trust in the CHWs. The presence of a doctor with CHWs during CoP sessions also added validity. Delivery of CoP sessions was perceived to be professional, especially since they were endorsed by local authoritative sources such as the municipality.

*WHC, CoP, SSI, CHW:* “*People are trusting me because I tell them that the information I have is not from any place, it is from the training I had with the American University of Beirut, and the Mobile University of health is a credible source for them.*”

#### 3.1.4 Knowledge acquisition & skillsets

Analysis of the knowledge assessments revealed a significant increase between the pre-tests and post-tests on all three health certificates (See Table 5) and most learners agreed that their knowledge and competence increased following the training, as reflected in the course evaluations. This was corroborated by the interviews conducted with the CHWs, where they re-iterated the increase in health knowledge following the CB training. This was especially the case for topics such as childbirth, pregnancy, gender-based violence, mental-health disorders, heart disease, cancer, among others. Information communicated in the certificates was relevant to people around them, as many expressed living with multiple family members who have NCDs. CHWs also highlighted increases in knowledge in topics that are related to children, such as child mental health and dietary needs, leading to improvements in parenting behaviour and skills. The MHPSS certificate also equipped them with essential counselling skills that they could put into practice when communicating their knowledge within the community.

*WH, CoP, FGD, CM.* “*I mean, even how we deal with the children changed a lot, it changed 80%, I mean for the silliest reason we used to get angry and hit the child, after the lessons and explanations that we too, we became more patient, for example we ask the child what is bothering him, so we don't cause psychological distress like we have*”*.*

CHWs acquired the ability to recognize symptoms in themselves and in others, which deepened their understanding and perception of community members' experiences. There was also a noticeable shift in the way they perceived mental health and the stigma associated with it, as they became more accepting of mental health disorders and openly discussed them. Another notable change communicated was their increased knowledge on the importance of prevention and early detection of disease through the women's health and NCD certificates.

*NCD, CB, SSI, CHW.* “*I always say it to others, to run tests on themselves, always so that the disease does not develop, and it would be in its earlier stages.*”

##### 3.1.4.1 Enhancement of interpersonal skills

The training has been reported to improve CHWs' basic communication and active listening skills. This new skill set helped CHWs to identify individual needs, build relationships with community members, and provide needed psychosocial support successfully across age ranges.

##### 3.1.4.2 Psychological improvement on the personal level

CHWs highlighted their personal growth as they gained increased self-awareness, allowing them to better regulate their stress levels. They also reported that they were better able to handle stressful events and to manage their reactions.

*MHPSS, CB, SSI, CHW.* “*When I participated in the training, I learned more about myself, and I know how to deal with my own self now*”

Following the CoP sessions, CHWs noticed improvements in their comprehension of the subject at hand. Leading the sessions themselves rather than being learners helped them gain a deeper understanding of the content, which assisted with long-term retention. They believe that this also increased their confidence and desire to disseminate information to the community.

*MHPSS, CoP, SSI, CHW.* “*You need to understand I better, you need to talk about it, give the information more time and know how to deliver it. I felt like I understood the topic better.*”

The CoP sessions increased community members' knowledge on health-related issues and clarified many misconceptions they had regarding diseases, women's health, and mental health issues. Many community members felt equipped with essential information promoting their ability to take care of themselves and others and they became aware of the significance of targeting health issues and the importance of seeking professional help, as well as new knowledge on what services are available in their areas. This indicates a strong resonance between the community members' perceptions following the sessions and their actual experiences as most community members agreed that they gained useful information and expressed confidence in their ability to apply the material to improve their health in the CoP survey.

##### 3.1.4.3 Attitudinal changes in CHWs and community members

There was a substantial attitudinal shift among CHWs and community members who attended the MHPSS initiative. CHWs and community members were more likely to advocate for mental health and the eradication of stigma surrounding it. Learners and members gained greater knowledge mental health issues and on the relationship between physical and mental well-being. This acquired knowledge shifted their perception of mental health and seeking psychological services and encouraged them to have positive attitudes toward seeking care. Some members of the community reported that they were more open to discussing mental health, facilitating referrals to providers without the fear of repercussions due to societal misconceptions and stigma. Finally, members were more likely to be understanding and open to needs of those struggling based on the information they received and learned in the CB and CoP sessions.

*MHPSS, CoP, FGD, CM:* “*Before they used to say if you want to visit a psychologist it means you are going to a doctor for crazy people, the idea was wrong and people saw it as a negative thing, now we accepted the concept and we are helping the community and applying it*”*.*

#### 3.1.5 Health-related behaviour

CHWs reported gaining useful competencies during the course evaluations immediately following the training. These changes were also communicated after the initiative and during the interviews. CHW had an enhanced capacity to address health-related concerns within their circles and were better equipped to provide advice on certain diseases, habits, and symptoms. They also actively engaged in discussions on common health subjects to dispel myths and share their knowledge with their communities. Their newfound interest encouraged them to access more information through various online sources. They reported changes that are related to their physical activity, eating habits and other things they are doing on a personal level including engaging in self-care to alleviate psychosocial distress. This was perceived as a positive factor by community members attending the sessions as it is considered modelling behaviour. Moreover, they noted an improved ability in referring people to services and a heightened understanding of others' conditions.

*MHPSS, CB, FGD, CM.* “*After she (CHW) benefited from the training, I started seeing her interaction with her kids, and it caught my eye, she told me that's what we took in the training*”

Through the CoP sessions, community members had access to this information and expressed a notable decrease in sugar intake, as well as dietary changes to mitigate the risks associated with certain health conditions. They also reported an increase in physical activity after learning more about its importance in prevention and management of certain NCD's. Self-examination practices display a better sense of awareness on women's health as well, underlining the successful delivery of knowledge about the importance of early detection. Another outcome of the initiative according to community members is their active involvement in their diagnosis and medical case, rather than passively receiving information only, and the sessions empowered them to make informed decisions about their health and treatment. This was also attributed to the marked increase in the ability to discuss health related topics and personal medical conditions. The pivotal role of CHWs was affirmed as community members continued to consult them for medical issues after the CoP sessions, portraying their reliability and the community's trust in the initiative.

*NCD, CB, FGD, CM.* “*We are more careful about salt intake, we know how to balance it, my mom at home has diabetes, now I know how to balance sugar with fruits and vegetables*”

Both CHWs and members of the community spoke of the effort they now put into seeking medical care and advice from healthcare professionals rather than relying on non-professional and outdated beliefs. The trend of delaying visits to professionals and neglecting their own health decreased and this was attributed to the awareness of the importance of early detection, intervention, and the ability to notice symptoms at an early stage. SSI's and FGD's revealed that for many participants, health has become more of a priority, and they are able to discuss their health openly as the environment has become more encouraging and safer due to the collective empowerment of health education in the community.

*WH, CoP, FGD, CM.* “*it has been 3 years since my last visit to a doctor specialized in women's health. Recently, after that* ‘*CHW*’ *spoke to me, I decided to visit [the doctor] and now for example I must do mammography […]. After listening to* ‘*CHW,*’ *I was encouraged.*”

#### 3.1.6 Individual and community empowerment

The MUH initiative transformed CHWs perception of themselves and their potential within their communities. The training sessions increased their self-confidence and their belief that they can provide the support and meet the needs of their community. They expressed the ability to provide vital assistance to community members and this endowed them with a profound sense of responsibility and purpose to continue educating people about health-related issues.

##### 3.1.6.1 Psychological improvement and social cohesion

The active participation in the CoP sessions and in a formal setting gave CHWs the strength to carry this information outside the training and increase their drive and impact within their community. They were perceived as knowledgeable and dependable by community members, having acted like role models in terms of healthy practices and changes, encouraging others to adopt similar habits. Their personal psychological well-being improved, leading to enhancements in physical and social functioning. This progress extended to family and community dynamics, reflecting their positive influence.

##### 3.1.6.2 Career and professional development

Following the positive changes in their attitude, knowledge, and leadership skills, CHWs expressed their increased drive to pursue further education and opportunities. It spurred them to organize meetings outside the ones organized by GHI and create spaces for community members to discuss health concerns and challenges. It encouraged them to reach out to women who were not able to attend CoP sessions and to plan support activities for children based on the knowledge they gained. The influence on professional development or impact extended beyond the CHWs and community members were also inspired to share knowledge within their wider community.

*WHC, CoP, FGD, CM.* “*I benefited a lot from the CoP activities, at first, I was a learner, but now I am the one responsible for leading the activities. I learnt how to explain and communicate the information and persuade others. I really benefited from being able to practically apply what I learned.*”

#### 3.1.7 Significance of CoP program

Findings of the qualitative data underscore the significance of the initiative, particularly the addition of the CoP component. Based on the interviews and FGDs, CoP sessions were perceived as highly beneficial for community engagement, with trained CHWs expressing strong belief in the value and significance of the program. One contributing factor was the alignment of the session content with the needs of the community, as community members also expressed satisfaction with the presented topics, emphasizing their relevance to their daily lives. This confirms the quantitative findings, showing that most community members agreed that the covered topics were convenient to the needs of their communities.

Beyond individual outcomes and personal well-being, several CHWs and community members reported that the initiative strengthened family and community systems, fostering greater connectedness and unity. Participants reported that the impact of the initiative also manifested in personal relationships within families, friendships, and the broader community, highlighting the positive impact on community welfare. Other benefits were reported, including heightened self-esteem, confidence, and knowledge, all of which are essential aspects of empowering communities and helping them communicate their needs to professionals confidently.

*NCDs, CoP, FGD, CM:* “*We have become, maybe even more confident in ourselves, in our information, we can have a conversation with the doctor*”

CHWs expressed appreciation for the efforts of GHI in organizing the trainings, and specifically for addressing issues related to mental health as it was reported to have improved overall well-being and acceptance within the community.

##### 3.1.7.1 Factors encouraging access to healthcare services

Several factors contributed to improving the community's motivation, awareness, and trust in accessing healthcare services, as highlighted in the data. For instance, CHWs and community members' awareness of existing health services within the community emerged as an important motivator to seeking care, in addition to availability of affordable health services. Knowing that certain health services are available served as a catalyst for people who might otherwise deter from seeking healthcare because of costs or proximity of services.

Knowledge increases of health-related problems is an outcome that encouraged access to healthcare as community members can recognize their symptoms and when they need professional help. Holding a certificate from a reputable organization like the American University of Beirut fostered trust between CHW and community members and increased receptibility of the information.

CHWs' role appeared to be a catalyst to accessing to healthcare services as they regularly interact with community members, provide guidance, follow up, and referral to the available services. Multiple CHWs expressed that they would sometimes accompany people to their appointments as a form of support, and this was perceived by community members as genuine interest in their well-being, leading to improved health outcomes.

*WH, CoP, SSI, CHW.* “*I help them when they refer to me for the smaller things. If a woman wants to go to the hospital or the doctor I accompany her, and we both benefit from the experience.*”

### 3.2 Challenges

Challenges of the CB and CoP sessions were grouped into four themes: (1) learning modality, (2) CoP logistics and delivery, (3) community response, (4) barriers to accessing healthcare services.

#### 3.2.1 Learning modality

Despite the value of having booklets to refer to, CHWs expressed that they did not contain all the information provided in the session by the instructor, which in turn affected their preparation and retention of information.

Some learners expressed the need for more elements that will maintain their attention in the CB component, as practical elements in videos were limited and instead the focus was on the theoretical content and written words. A recurring opinion amongst CHWs completing all three certificates was the need for longer hours to cover a broader spectrum of information and allow for more in-depth discussion. Despite content alignment between the CoP and the MUH training, attendees expressed a desire for more elaborate discussions in the CoP sessions.

*WH, CoP, SSI, CHW.* “*I wish the duration of the session was longer. We (CHWs) were giving 5 topics in 2 h, sometimes it felt like the topic was not taking the time it deserves, especially for those who want to ask questions. Time was chasing us, and it should have been longer.*”

*MHPSS, CoP, FGD, CM.* “*The cycle was nice but as the other lady said before, you feel like it needs more time, but it was nice, and it motivates you to attend again*”

#### 3.2.2 Cop logistics and delivery

Opinions regarding the duration of the CoP sessions were mixed, with some expressing the need for more time, and some community members finding the sessions excessively lengthy, reflected in both the interviews and the survey results showing some community members not agreeing that the duration of the event was appropriate. Some CHWs needed to summarize their presentations and information due to time constraints and wished for more discussion time. There was a difference among CHWs in terms of public speaking and session delivery level, with some showing nervousness, leading to challenges in information sharing as well as moments of hesitancy when responding to questions posed by community members. This was initially seen in the feedback provided by the community members in the survey, specifically in the MHPSS CoP sessions. This was acknowledged by the CHWs themselves as some expressed the need for more training and preparation before the sessions to improve this issue. Enhancing long-term retention of learned material proved difficult without efficient follow-up post-training.

*NCD, CoP, FGD, CM.* “*You said to be honest, not all of them (were good at presenting), there were two ladies that I could not even hear, they were nervous.*”

Additionally, the training schedule posed challenges for the community members, especially mothers who have homes and children to attend to. The location was also brought up as an issue for both CHWs and community members due to difficulties commuting. Negative feedback emerged regarding bringing children along to the sessions as they cause distractions and disruptions throughout the sessions. Some CHWs found the physical space uncomfortable and small considering the number of community members attending and the time spent there.

*WH, CoP, SSI, CHW.* “*My first comment would be the location; it was very small and noisy [….] I am talking about the women who attended from my end and what they told me, and they did not like this, and they also did not like the duration, it was too long for them (the session).*”

#### 3.2.3 Community response

Some resistance and lack of receptiveness were encountered when CHWs attempted to share mental health support, specifically when advising on seeking professional psychological help. This finding is highlighted in the results of the CoP survey conducted for the MHPSS sessions, where quantitative analysis revealed lower scores in participants' belief that they could apply the material to improve their health, as compared to the other health topics. This stemmed from the stigmatization of mental health challenges and the community's perception of it, making it a barrier to accessing care. There was also a lack of understanding on what the CHWs role is exactly, and this hindered their relationship with community members and the ability to establish trust in their advice.

*MHPSS, CoP, FGD, CM.* “*Sort of you can say they could not convince me in a way, I was not convinced that if the symptoms happened to me that I would go to a psychologist or do this or that (techniques provided by CHW).*”

#### 3.2.4 Barriers to accessing healthcare services

Limited awareness of available healthcare centres and services presented as a challenge for people who did not attend the CoP sessions. Specifically, psychological services and psychotherapists were not widely available in remote areas, especially coupled with transportation constraints. As previously mentioned, the initiative increased knowledge on the need to seek medical care and the availability of the services, however, financial barriers deterred individuals as free healthcare is rare and they would still need transportation fees and medication costs. Aside from this, participants communicated to presence of long waiting lists and slow follow-up, demotivating individuals who want to seek help. One disadvantage the CHWs faced was their lack of formal affiliations with organizations that could help them approach NGOs for referrals.

*WH, FGD, P2:* “*When I am chatting with people, they tell me that doctor fees and medications are expensive. This is why they become demotivated to see a doctor*.”

Given the nature of the communities the training is provided in, gender-specific barriers were an issue with women unable to visit doctors due to spousal refusal and the general lack of knowledge and awareness among males. Some women also expressed that they would prioritize their children's health over their own, thus overlooking their own symptoms.

*WH, CoP, FGD, CM:* “*For example, there are things that I would like to do but my kids are a priority and sometimes I neglect myself for my kids.*”

## 4 Discussion

As the deterioration of Lebanon's healthcare system has had a significant impact on its ability to meet the healthcare needs of its population (7), there is an urgent need for action to provide health services, basic knowledge, and improve access, especially in resource-limited settings with shortages of healthcare workers and educational resources (21, 22, 53). Existing literature highlights task-shifting as a tool to bridge health access gaps in fragile settings (38, 54), and CoPs as serving to spread public health knowledge efficiently within communities (35, 36). Building upon the existing literature and recognizing the scarcity of research on GHCB initiatives in the MENA region (40), this study aimed to evaluate the long-term impact of GHIs MUH initiative. MUH aims to empower CHWs with targeted health-related skills and knowledge, to assist in alleviating the strain on the healthcare system by strengthening community-based healthcare and health knowledge. Over 3 years, the program implemented training on women's health, MHPSS, and NCDs. Employing a mixed-methods approach, this study examined both quantitative and qualitative data to assess the short and long-term effectiveness of the program on both individual and community levels. Following the pilot implementation of the first MUH cohort (41), a CoP component was introduced based on identified gaps to enhance the dissemination of knowledge within the communities and to equip CHWs with more practical and leadership skills.

Consistent with the finding of the pilot MUH program, this study demonstrates in a general sense, positive impacts of the initiative on health-related knowledge, attitudes, and behaviours of CHWs and community members. This serves as substantial support and validation of the success of MUH as it examines a larger sample size, and it covers a wider array of health topics yielding similar results. Importantly however, it also incorporated the CoP component, enabling CHWs to conduct sessions within their communities, and allowing for a more comprehensive evaluation of the initiative.

Learners expressed that they received novel and important health-related knowledge and education that they would have otherwise not been exposed to in their communities. This stresses the importance of the mobile approach in improving access of vulnerable communities in Lebanon to health. Furthermore, high satisfaction rates were reported in course evaluation surveys as with the pilot study, emphasizing participants' positive perception on the quality of the training and suggesting that the topics were aligned with their needs.

The CB component provided CHWs with knowledge on health topics through diverse learning modalities, including videos, visuals, and interactive discussions. This approach seemed to resonate with learners, helping them better understand and retain the information. The use of technology, such as through laptops and videos, also equipped them with valuable tech-related skills, which they reported to be useful for future independent learning. Quantitative results revealed a significant increase in scores on pre-post knowledge assessments across all three health certificates. This was also corroborated by qualitative data highlighting that the training effectively increased CHWs knowledge and positive health behaviours. This is an important outcome of the initiative because education on health-related topics has an influence on health beliefs (55). Despite the success of the training, CHWs reported difficulty with long-term retention of information, consistent with findings of the pilot implementation and previous literature on training of CHWs (41, 56, 57). There were also limited opportunities for dissemination of information following the CB training, as CHWs relied on informal and indirect contact with friends and family members within their environment.

The qualitative analysis of interviews and FGDs further validates the initiative's success, revealing the pivotal role of the CoP component in enhancing effectiveness and achieving the goals of the initiative when compared to the stand-alone pilot implementation. The CoP sessions emerged as an important facilitator for long-term retention and better comprehension of materials learned in the CB training. This was reported by several CHWs, emphasizing that the experience of delivering the information to an audience helped them acquire a deeper and better grasp of content. Sharing of personal experiences and in-depth discussions during the CoP could have also been a factor for better retention of information (58). Delivering the material through different platforms was especially appreciated in the CoP component, as it assisted CHW in presenting the information effectively, which is consistent with feedback from CHWs in other initiatives (59). Blended learning also kept community members engaged throughout the sessions, which was supported by the high on delivery of information in the CoP satisfaction survey.

The CoP sessions appeared to be a key element of the initiative as they provided CHWs with a formal opportunity to disseminate knowledge, giving them direct contact with community members and an opportunity for professional development, which may improve their chances for future employment. This added value to the initiative by overcoming an important gap identified in the pilot, in which CHWs were reported to have limited necessary skills to serve as advocates within their communities. At the individual level, the CB with the supplementary CoP component enhanced the overall learning experience of CHWs, improved their interpersonal skills, confidence, and gave them a stronger sense of purpose within their communities. A keen sense of empowerment was established among CHWs, making them feel responsible for disseminating knowledge and acting as role-models within their communities. This has been addressed in other programs engaging CHWs, reporting that they felt empowerment and a sense of accountability to their communities (60). Another aspect that contributed to the effective execution of the CoP activities was the assistance and constructive comments that CHWs received from the facilitators, as well as the presence of an expert doctor who gave professional support and insight. This increased the legitimacy of the CoP operations and enabled CHWs to play a larger role in disseminating health-related information. The ability to apply their knowledge in the CoP sessions helped community members perceive them as professionals and a credible source of information, becoming trusted members of their communities. For example, they were more confident in providing community members with information regarding available services, referring them to doctors and accompanying them at times.

A principal factor that was noted in the data is that CHWs encouraged community members, specifically women, to take an active role in their health. CHWs worked on reframing women's traditional values to prioritize self-care and health maintenance by having conversations about women's health with their daughters discussing sensitive topics within their communities. Training women to become active CHWs within their communities through CB and CoP led to better adoption of health habits throughout the wider community, as they supported them in building relationships with community members, guiding them, and following-up with them regarding health issues. This aligns with existing literature on CHWs role as facilitators of health-related behaviours (61), and the success of CHW programs in promoting positive health changes as well as screening for diseases (62). Our results underline the positive change in health behaviours in the community as reported by both CHWs and community members although our data sources relied on self-reported narratives. The reported changes included physical activity, dietary changes, ability to recognize symptoms, and higher willingness to seek medical treatment. CHWs and community members equally reported that they incorporated new health related behaviours and self-care habits for themselves and family members, supporting their response in that they believe they are able to apply the information they learned to improve their health, as reported in the surveys Additionally, there was a notable emphasis on prevention and early detection with CHWs encouraging prompt access to health services and frequent testing, particularly for issues like heart disease and cancer. Some community members reported that they became aware that early detection could contribute to better health outcomes in the long-run and started addressing their health needs more promptly. Early diagnosis and control of NCDs has been linked to better health outcomes and a decrease in cost of health services and the need for tertiary treatment (15), which aligns with the goals of the MUH initiative in addressing the gap in health needs and health access.

There were several important changes on the community level that further validate the success of MUH in addressing health needs of the community. There was an increased trust in CHWs after the CoP sessions, with community members acknowledging the value of information being communicated to them by the CHWs. This finding was consistent from the time of the CoP sessions until months after when discussed during the FGDs, revealing long-term changes in the relationship between CHWs and community members. This is a particularly important finding, as trust in CHWs and health systems is linked to better health outcomes as well as increased odds of accessing health services again (63, 64). Evidence shows that CHWs play a significant role in patients increased adherence to care, and higher engagement with health services (65, 66). In our study, community members perceived CHWs as knowledgeable, supportive, and capable of addressing their health-related inquiries.

Moreover, the CoP component, specifically within the MHPSS initiative, was stated to have contributed to reduced stigma surrounding mental health issues within the community. Mental health has been a topic that was previously stigmatized and surrounded by many misconceptions, some of which have been clarified through the CB and CoP sessions. There was a reported increase in unity and social connectedness within the community, also reflected in relationships within families and friends. This in turn supported better dissemination of knowledge and support for medical issues and health seeking behaviors. Families and friends are considered frontliners for support, especially regarding mental health issues among vulnerable communities (26). An increase in social connectedness, which was triggered through MUH, can increase refugees' comfort, knowledge, and trust in healthcare systems, and can potentially improve wellbeing and health outcomes (67). Positive community feedback on the relevance and accessibility of CoP sessions underscores their significance in providing a platform for dissemination of health knowledge and empowering communities. The CoP sessions provided a safe space for participants to discuss sensitive and personal experiences, aligning with previous literature indicating that creating a safe and inviting space can help decrease isolation, strengthen relationships, and enhance knowledge and practices (68), with potential broad community level impact as demonstrated in this study.

Notwithstanding the initiative's success in conveying health education and bridging a gap between health needs and contact with the healthcare system, ongoing challenges persist, hindering access to services. This is consistent with the findings of the first pilot implementation and is intricately tied to the unique context of Lebanon. For instance, feedback from some CHWs and community members indicated that cost remains a significant barrier to accessing health treatments, particularly for specialized treatments, and especially in remote areas where financial resources are limited, and travel to distant facilities is often impractical. Women are more likely to prioritize providing basic survival needs for their families at the expense of taking care of their own health whereby they may defer seeing a doctor in order to ensure available resources are utilized by other family members, especially their children. Consistent with the literature, stigma remains a barrier in accessing health services, especially in relation to mental health, which remains largely a taboo and misunderstood topic (69). Gender-specific obstacles were also noted as some women encounter resistance and refusal from their spouses when planning to seek essential healthcare services. This is due to cultural norms against girls visiting women's health doctors in addition to lack of awareness on the significance and potential consequences of health-related issues.

In addition, the CoP component presented its own set of challenges, mostly in relation to varying opinions on the length of the sessions. Aligning with the results of the CoP satisfaction survey on the length of the sessions, some community members found the sessions too short to cover the necessary information, while others found them lengthy, which interfered with their responsibilities at home as caregivers. Results revealed the potential need for additional training prior to CoP sessions as some CHWs reported experiencing nervousness, difficulties with public speaking and challenges in answering certain questions during the sessions, as they had not led similar activities before. This was also perceived by a few community members in FGDs and reflected in the feedback provided after the CoP session, emphasizing the need to have more suitable CHWs leading the sessions. Although varying presentation and communication skill sets are expected in such an initiative, more effort should be placed on better preparing CHWs to lead CoP sessions and building their self-efficacy accordingly. Moreover, the session location was not favored by some cohorts due to distance from their homes and surrounding noise levels, along with other distractions like attendees brining their children to the session, potentially hindering engagement and understanding.

### 4.1 Limitations, recommendations, and conclusion

While our study provides valuable insights into task-shifting and CoP with under-served communities, it is essential to acknowledge several limitations that may affect the interpretation of our findings. First, given that the data collected for the surveys was self-reported, it introduced the possibility of social desirability, which may have influenced participants to provide responses they deemed socially acceptable. Moreover, the CoP evaluation survey administered by evaluators from GHI, may have heightened this bias as participants may have responded based on what they felt aligned with evaluator expectations, especially that this may have been perceived as humanitarian service to their community. Another factor that may have contributed to the high scores on these surveys was the fact that most of these women may have not participated in similar activities before, thus had no baseline to compare the trainings to or accurately evaluate them. Secondly, the absence of quantitative measures for changes in health behaviors and outcomes is an important limitation restricting our ability to objectively assess the larger impact of the program. The study relied primarily on qualitative data from the perceptions of CHWs and community members.

Several recommendations were established based on the findings of this study, which will help tackle the limitations observed as well as improve on future initiatives in similar contexts:

➢ To mitigate the potential impact of social desirability bias, it is suggested to give community members the opportunity to answer the surveys themselves whenever possible, following the CoP sessions. This may yield more accurate responses as compared to being asked the questions by the evaluators.
➢ Creating child-friendly spaces for children during the CoP sessions can tackle challenges with distraction and facilitate attendance for women with family commitments.
➢ Allocating more time for CoP sessions may be advantageous in future initiatives to allow CHWs enough time to cover necessary materials and to extend discussions as they were found to be major components when it comes to knowledge acquisition and engagement.
➢ Introducing further training or short sessions focused on public speaking and presentation skills can enhance CHWs abilities to effectively lead CoP sessions, tackling a challenge that was noted in our analysis.
➢ Refresher courses for CHWs may be beneficial to support long-term retention and encourage application of acquired knowledge. These courses can be shorter and accommodate more participants, or can be in the form of pre-recorded videos or new health information to promote continued learning and practice.
➢ Establishing long-term and continued communication channels between CHWs and community members can ensure the sustained success of the initiative, fostering ongoing support and empowerment within the communities.
➢ CHWs called for more initiatives covering additional topics such as first aid training, and community members expressed enthusiasm to attending them and mentioned the need for even more CHWs. Thus, it is crucial to secure more funding to ensure sustainability of this work to reach more CHWs across wider low-resource and vulnerable communities in Lebanon. It is also worth exploring scaling MUH and replicating it in neighboring countries hosting large numbers of refugees such as Jordan and Turkey for example following needed contextual adaptations.

This study sheds light on the significance of MUH and its potential to improve health education and behaviors in vulnerable communities. MUH had a positive impact on women in the targeted communities, addressing crucial health needs. Particularly, the supplementary CoP component emerged as a powerful tool in empowering CHWs and providing them with a formal platform to share their knowledge, transforming their role within their communities. The findings of the study showed positive individual and community level changes related to health knowledge, behaviors, skills, and empowerment. Through the program, this knowledge can continue to be disseminated within these communities through CHWs and community members who attended the CoP sessions. The initiative assisted in reducing stigma, fostering trust between CHWs and community members, leading to increased engagement with health services. Efforts to address the challenges revealed in this study should be prioritized in future initiatives and recommendations can be considered when tailoring future training. The comprehensive evaluation done in this study on a larger scale provides valuable insights on the initiative's effectiveness and serves as an example for implementation of similar initiatives in other countries to address health needs in the region.

## Data Availability

The raw data supporting the conclusions of this article will be made available by the authors upon reasonable request.
