# Supplementary material for: Capacity building and community of practice for women community health workers in low-resource settings: long-term evaluation of the Mobile University For Health (MUH)
Source: Front Glob Womens Health. 2024 May 20;5:1304954. doi: 10.3389/fgwh.2024.1304954 (PMC11144904; doi:10.3389/fgwh.2024.1304954)
Supplement: Supplementary file 1 [file Table1.docx]

| Tool | Questions |
| --- | --- |
| Semi-structured interview guide | 1. Describe your learning experiences during your participation in the training. |
|  | 2. How did the [online/blended/face-to-face] learning modality influence your learning process? |
|  | 3. Describe your knowledge in ___ after your participation in the training. |
|  | 4. Describe your practices in ___ after your participation in the training. |
|  | 5. How did the training impact your capability to learn new skills? |
|  | 6. What are the strengths and weaknesses of the training program? |
|  | 7. Explain whether the certificate on___ , prepared you to respond to your community’s health needs as a community health worker. |
|  | 8. Since your participation in the ____ training, please describe your experience referring members of your community to receive healthcare services. |
|  | 9. 3. Describe the extent to which your contributions as a community health worker meets the expectations of your community members in terms trust and quality of care. |
| Focus group guide | 1. Describe your and your community’s experiences in accessing _________healthcare services? |
|  | 2. To what extent do you trust the healthcare services delivered by the community health workers  regarding ____ after the training? |
|  | 3. Describe the availability and quality of healthcare services regarding _____ in your community after  the training. |
|  | 4. To what extent are the trained community health workers responding to the health needs of your  community regarding ___. |

Appendix 1. Courses covered under each certificate.

|  | Certificate | | |
| --- | --- | --- | --- |
| Topics | ***Women’s***  ***Health*** | ***Mental Health and Psychosocial Support*** | ***Non-communicable***  ***Diseases*** |
|  | Maintaining Women’s Health | Introduction to Mental Health | Nutrition and Healthy Lifestyle for Prevention and Control of Chronic Diseases |
|  | Reproductive Health | Mental Health Disorders | Cardiovascular Diseases |
|  | Pregnancy | Mental Health and Sensitive Cases | Cancer |
|  | Older Women’s Health | Children’s Mental Health | Pulmonary Diseases |

Appendix 2. Capacity Building SSI and FGD Questions

Appendix 3. CoP SSI and FGD Questions

| Tool | Questions |
| --- | --- |
| Semi-structured interview guide | 1. Describe your overall experience during your participation in the activities. |
|  | 2. How did these events and activities advance your learning process? |
|  | 3- Describe your knowledge in ____ after your participation in the activities. |
|  | 4. Describe your practices in ____ after your participation in the events.   - 4.1- To what extent do you interact with your community members? - 4.2 Describe whether you feel prepared to address their health concerns. - 4.3 Since your participation in the ____ activities, please describe your experience providing basic health education and referring members of your community to receive health services. |
|  | 5. Describe your level of motivation to carry out the assigned duties?   - 5.1- What would you consider as motivating/demotivating factors? - 5.2- What factors are considered to facilitate/hinder the process of implementing the activities? |
|  | 6. Comment on your assigned activities and responsibilities.   - 6.1- To what extent do you feel that the content of the activities was harmonious with the previous course material you received through MUH? - 6.2- To what extent do you feel that your assigned activities and responsibilities were clearly communicated to you by the supervisors? - 6.3- To what extent do you feel that you were well-prepared and properly trained to carry out the assigned activities? |
|  | 7. To what extent do you feel that community members trust your role as a community health worker, to provide them with good quality of health services? |
|  | 8- To what extent do you believe the work you’ve done in the community will result in positive outcomes?   - 8.1- What factors would you consider as facilitators/barriers to implement these outcomes? |
|  | 9. In your opinion, what are the strengths and weaknesses of these activities? |
| Focus group guide | 1. Describe your and your community’s experiences in accessing services or information related to ____. |
|  | 2. To what extent do you believe the CHW improved your access to healthcare and your  health-related knowledge? |
|  | 3. Describe whether you interact with the CHW on day-to-day basis and to what extent is she helpful in answering your health-related concerns. |
|  | 4. To what extent do you believe you were able to apply what you learned in the activities?   - 4.1- What factors would you consider as facilitators/ barriers to applying what you learned? |
|  | 5. To what extent do you trust the health services delivered by the community health  workers regarding ____ after attending the activities? |
|  | 6. To what extent did the trained community health workers respond to the needs of your  community regarding ____? |
|  | 7. To what extent did the CHW increase your self-efficacy and confidence in managing your health-related issues i.e. change your health behaviors following your participation in the event; increase your confidence in asking your doctor questions related to your health condition? |
|  | 8. To what extent do you feel that direct engagement with CHWs allowed you to enhance your knowledge and skills regarding ____? |

Appendix 4. Course Evaluation Survey Results. Data is presented as % of participants who agreed or strongly agreed with the above statements

| **Evaluation Component** | **Learner’s evaluation** | | |
| --- | --- | --- | --- |
|  | Women's Health (n=27) | Mental Health and Psychosocial Support (n=27) | Non-communicable Diseases (n=29) |
| *Course Delivery* |  |  |  |
| The course objectives were clearly stated or presented. | 100* | 100 | 100 |
| The content was relevant to my needs. | 89 | 100* | 97 |
| The course objectives were fulfilled. | 100* | 93 | 100 |
| I would take another course via this delivery method. | 100 | 100 | 100 |
| The allocated time for the course was appropriate. | 100 | 100 | 97 |
| The pace of the course was appropriate. | 100* | 100* | 97 |
| *Course Content* |  |  |  |
| The course content was organized and easy to follow. | 100 | 100 | 97 |
| My knowledge and competence in this subject matter increased. | 100 | 100 | 100 |
| I have gained useful competencies from this course. | 100 | 100 | 100 |
| I am satisfied with the overall quality of this course. | 100 | 100* | 97 |
| I would recommend this course to others. | 100 | 100 | 100 |
| I am willing to participate in future courses held by GHI. | 100 | 100 | 100 |
| *Instructors* |  |  |  |
| The instructor was well prepared and knowledgeable about the topic. | 100 | 100 | 100 |
| The instructor communicated information clearly and effectively | 100 | 100 | 100 |
| The instructor stimulated interest in this subject | 100 | 100 | 100* |
| I am satisfied with the overall performance of the instructor | 100 |  | 100 |
| * Missing one response |  |  |  |

Appendix 5. CoP Community Members Evaluation Survey Results

| **Evaluation Component** | **Women's Health (n=119)** | | | | **Mental Health and Psychosocial Support (n=38)** | | **Non-Communicable Diseases (n=54)** | |
| --- | --- | --- | --- | --- | --- | --- | --- | --- |
|  | **Beirut** | **Akkar** | **Bar Elias** | **Bourj Hammoud** | **Tyre** | **Haret Hreik** | **Haret-Hreik** | **Chouf** |
| **Activities’ Topics** |  |  |  |  |  |  |  |  |
| The health topics covered are convenient for the needs of my community. | 96 | 73 | 94 | 100 | 100 | 100 | 100 | 91 |
| The material was organized and easy to follow. | 100 | 100 | 97 | 100 | 94 | 96 | 97 | 100 |
| I have gained useful information from attending these events. | 96 | 93 | 97 | 100 | 94 | 100 | 100 | 96 |
| I would recommend these activities to others. | 100 | 100 | 100 | 100 | 100 | 100 | 100 | 100 |
| I am willing to attend future events. | 100 | 83 | 97 | 91 | 100 | 96 | 100 | 100 |
| I am satisfied with the overall content of the material. | 100 | 98 | 100 | 100 | 94 | 100 | 97 | 96 |
| I believe that I am able to apply the material I learned to improve my health. | 100 | 100 | 94 | 100 | 69 | 77 | 94 | 87 |
| **Delivery Method** |  |  |  |  |  |  |  |  |
| I believe that these activities will allow better access to healthcare services in the future. | 100 | 90 | 94 | 100 | 100 | 86 | 100 | 91 |
| Interacting and discussing with CHWs fostered learning and knowledge sharing. | 96 | 90* | 97 | 100 | 94 | 100 | 94 | 96 |
| Interacting and discussing with CHWs increased my knowledge in the subjects covered. | 100 | 85 | 100 | 100 | 81 | 100 | 100 | 100 |
| The educational material (pamphlets, posters...) facilitated the delivery of material. | 100 | 100 | 100 | 100 | 88 | 100 | 100 | 96 |
| The duration of the event was appropriate. | 100 | 93 | 97 | 100 | 94 | 86 | 77 | 87 |
| The CHWs stimulated interest in the subject of the topic. | 100 | 100 | 97 | 100 | 94 | 100 | 97 | 100 |
| **CHW Performance** |  |  |  |  |  |  |  |  |
| I trust that CHWs are well-trained to address the health needs of my community. | 100 | 100 | 97 | 100 | 94 | 96 | 74 | 87 |
| The CHWs were knowledgeable on the topics they presented. | 100 | 90 | 97 | 95 | 100 | 96 | 87 | 83 |
| I am satisfied with the level of engagement and interaction with CHWs. | 100* | 98 | 100 | 100 | 63 | 96 | 94 | 100 |
| The CHW was friendly and supportive. | 100 | 98 | 100 | 100 | 100 | 100 | 100* | 100 |
| The CHW was respectful and non-judgmental during the implementation of the event. | 100 | 98 | 100 | 100 | 100 | 100 | 100 | 100 |
| The CHW understood my culture and values. | 96 | 88 | 100 | 100 | 100 | 100 | 100 | 100 |
| The CHWs translated and tailored positive health behaviors and health promotion strategies that might encourage you to adopt healthy behaviors in the future. | 96 | 73* | 100 | 100 | 94 | 96 | 97 | 100 |
| I am satisfied with the overall performance of CHWs. | 100 | 100 | 100 | 100 | 94 | 100 | 100 | 100 |

* Missing one response
