# Supplementary material for: Capacity building and community of practice for women community health workers in low-resource settings: long-term evaluation of the Mobile University For Health (MUH)
Source: Front Glob Womens Health. 2024 May 20;5:1304954. doi: 10.3389/fgwh.2024.1304954 (PMC11144904; doi:10.3389/fgwh.2024.1304954)
Supplement: Supplementary file 2 [file Datasheet1.pdf]

Appendix 6. Results of qualitative analysis: emerging themes and codes

Category	Theme	Code
**Strengths**	1. Access to Education	Exposure to new, relevant, and essential information on health-related topics
		Offered attending women new opportunities for education and increased willingness to learn more about health issues
		New educational experience of receiving information through videos, PDFs, and visuals in a mobile classroom environment
		Expressed interest in joining future training initiatives by CHWs and community members
		COP activity sessions were accessible to women from different educational levels and different locations
		Community members expressing the need for more CHWs and similar initiatives considering the benefits of these trainings
		Community member pointing to the significance of maintaining communication with the CHW post the awareness activities to facilitate the exchange of knowledge and ideas
		Novel insights that clarified misconceptions about health-related issues.
	2. Learning Modality	Availability of videos, visuals, and PDF format helped the learners to receive the information in a clearer way and facilitated CHW's capability in presenting the material during the COP sessions
		Face-to-face interaction ensured exposure to various perspectives and ideas of other individuals in the room and allowed an increased involvement of learners of various ages and educational levels.
		Face to face modality allowed the practical experience of modeling behavior that needs to incorporated when interacting and communicating with others (e.g. proper usage of eye contact)
		Preference of shared real-life experiences, discussions and examples in comparison to content presented visually through the screens.
		The use of the computer during the training helped the CHW in developing the needed skills on how to use the computer and encouraged them to use it for learning purposes
		Lectures and videos helped simplify the learning process and make more comprehensible
		Lectures and videos attracted the learner's attention and enhanced engagement
		Interactions with the doctor and shared feedback was helpful
		Usage of laptop facilitated the learner's capacity to comprehend the information by allowing for easier follow-up with the instructor's explanation
		Blended learning promoted information clarity and comprehension by presenting the information form multiple resources (orally explained and visually presented) .
		The use of sample models was regarded as a highly effective learning approach when exploring the health systems related to women's health and NCD's
	3. CoP Session Logistics and Delivery	The information presented by the CHWs was regarded as sufficient, important and comprehensive
		The COP sessions were well-planned and well-prepared ahead of time, allowing CHWs to feel confident in their ability to present information and guide the sessions.
		COP content and materials were regarded as relevant to the community's mental health needs which increased community members engagement with the sessions and interaction with CHWs
		Community members with low education level were able to enrich the discussions and raise insightful questions during the COP awareness sessions
		Real life examples, emotional involvement, shared experiences and interactive questions among the community members improved comprehension, increased knowledge and widened perspectives
		The information being presented was experienced as enjoyable, informative and interesting.
		Subtheme: Feedback of Performance of CHWs during COP Sessions
		Positive feedback on the willingness of the CHWs and the doctor to answer and discuss any inquiries or questions
		Community members indicating that they received adequate support from the CHWs
		CHWs were seen to be confident when delivering the sessions and eager to spread the knowledge
		CHWs were understanding and were able to provide a safe space for the discussion of personal and sensitive topics.
		CHWs provided the community members with the motivation to learn the health-related information
		CHW was skilled at conveying knowledge in a way that emphasizes the most important ideas and persuades others to follow its guidance and put it into practice.
		CHWs provided guidance and advice that took into consideration community members' financial challenges.
		Subtheme: Trust in CHWs and Initiatives
		Community members trusted CHWs' advices and approached them for guidance after CHWs' attainment of certificate of training and conduction of the COP awareness sessions.
		Community members trusted information, materials and documents conveyed during the COP awareness sessions given its source from a reputable institution (American University of Beirut- AUB)
		The relevance of the community member's real-life experiences to the information being shared by the CHWs promoted the community member's trust in them.
		Growth and positive change observed in CHWs established community members' trust in the CHWs
		The community members considered the doctor's on-site presence, complementing the CHWs' explanations during the COP meetings, to be a source of credibility and trust.
		Professional efforts exerted to implement and coordinate the COP sessions added to community members' sense of trust in CHWs.
		Invitation to participate in the COP awareness sessions from credible sources such as the municipality established community members' trust.
		Subtheme: Interaction between CHWs and Coordinators
		Coordinators were supportive and readily available to answer questions, provide guidance and assistance and address any arising concerns.
		Assigned activities and responsibilities for trained CHWs were clearly communicated and constructive feedback was presented when needed.
		CHWs felt that the facilitators created a comfortable environment and maintained clear communication
	4. Knowledge Acquisiton and Skills	***At the CHW Level:***
		Increased knowledge in mental health topics such as anxiety, depression, substance abuse, aggressive behavior, dementia, parental skills / health related topics.
		Increased knowledge and ability in recognizing symptoms of self and others and engaging in referral procedures for proper treatment
		Acquisition of counselling skills

Ongoing dissemination of information to the community members and the application of the knowledge acquired during training to real-world circumstances and experiences allowed for long-term retention of information

Increased knowledge of what others of the community are going through, as well as increased capacity to comprehend their children's behavior and increased knowledge on how to treat them

Personal change in perspective on mental health and eradicating mental health misconceptions and stigma

Identifying people in need of immediate psychological assistance

Ability to detect that different type and sub-types of mental disorders

Increase in self-perceived competence in working with mentally unwell individuals.

Improved understanding of the importance of prevention and early detection of disease.

Subtheme: Enhancement of Interpersonal skills

Enhanced skills in basic communication and active listening

Engage in empathetic contact and supportive interaction/ Enhanced skills in relationship-building skills or empathic capacity

Increased ability to assess and understand the needs of individuals

Increased ability in listening actively and supportively to others//Improved ability to actively and supportively listen to others - increased listening skills (psychosocial support) regardless of age groups (children and adults)

Subtheme: Psychological improvement on the personal level

Self-awareness and ability to deal with own stress

Increased ability to control ones emotions and reactions to disturbing incidences

Post COP:

Improvement in knowledge and deeper understanding of health-related issues and topics, and increased capability to recognize various health conditions

Improvement in capacity and confidence to deliver health awareness and execute health care support

Increased knowledge on how to communicate and share knowledge and advice with others, specifically strengthened through leading COP sessions

CHW was able to understand training content more deeply and experience higher retention and acquisition of the information when conducting the COP sessions.

CHW reporting first time conducting awareness seminars in front of an audience, which increased their confidence in presenting the information and spreading knowledge later on to community members

At the community member level:

Increased knowledge on health promoted the ability to take care of self and others (family members, friends or acquaintances from the community)

Improved awareness on significance of targeting mental health issues and seeking professional psychological help.

Acquisition of new health knowledge following interaction with CHW (through group sharing of personal experiences, self-reflection, informal conversations) who were family members, friends, or acquaintances from the community

Medical misconceptions were addressed and rectified - such as correcting false information on menopause or obesity

Community members gained knowledge on the availability of centers that offer health services.

Post COP:

Community members developed the skills necessary to have a supportive [psychoeducative] conversation with children engaging in maladaptive behaviors and offering suggestions for alternatives healthy behaviors.

Improved awareness on the significance of seeking MHPSS services

Increased ability to detect mental issues and symptoms in the elderly, children and women

At the CHW Level and community member level - Attitudunal Change

Explaining to others the significance of targeting one’s mental health in an effort to eradicate the stigmatizing views

Understanding the role of distress and mental health issues in aggravating various medical diseases and/or serving as the underlying causes behind any psychosomatic complaints

Negative perceptions of MHPSS services have changed, increasing the community's acceptance of these programs.

Increased courage to discuss mental illness topics with others in the community and refer them to seek MHPSS services

Overall positive change in attitudes expressed by the CHWs and community members

CHWs reported increase in altruistic sentiment and the need to understand who are suffering from mental illness

5. Health-related Behaviours

At the CHW level:

Enhanced capacity and confidence to assist others with health-related issues by discussing common health subjects, sharing advice and knowledge, and dispelling misconceptions and myths to friends, family, and community members

Increased interest to learn more by navigating websites and other sources

Change in health-related behavior among CHWs at the personal level following sessions

Increased ability for self-care – engage in reduction of psychosocial distress techniques

Acquired capacity to identify, intervene on, and monitor mental health and psychosocial problems

Increased access to psychosocial services at the personal level

Ability to engage in referral practices

Engaging in relaxation and breathing exercises for alleviating stress and incorporating them into daily life

Acquiring new knowledge, improving thinking methods, and broadening thinking styles

Increased capacity to interact and form empathic connection to others

Increased capability to provide advices on parental skills

	Post COP:<br>Increased ability and confidence to referring children and adult community members to seek professional psychological support from available mental health services in the area<br>***At the community member level:***<br>Prioritizing mental health in their lives and experiencing encouragement in discussing personal health issues.<br>Engaging in referral of acquaintances in the community to seek mental health service and professional psychological support mental health practitioners<br>Increased access of psychosocial services<br>***Post COP:***<br>Improved self-reflection among the community members to understand their health status and adopt new behaviors to improve their health<br>Interactions and discussions with the CHWs during the COP activities allowed the community members to gain better awareness towards their children's mental health and avoid emotionally aggressive behaviors towards them.<br>Community member started engaging in healthy and stress releasing activities like exercising and having walks to improve their mental health amid the socio-economic stress factors.<br>Continued self-education after attending the training through navigating the internet and watching YouTube channels<br>Forming support groups and networks to provide psychosocial support to psychologically in need individuals in their neighborhood<br>Increased awareness on the usage of appropriate coping strategies when experiencing mental health and psychosocial problems - increased ability to cope with the everyday stresses in life and manage distress<br>Acquisition of new parenting skills and increased ability to handle and communicate with children<br>Engaging in less blame of others' negative behaviors and becoming more understanding of the underlying psychological causes behind others' unhealthy/negative behaviors<br>Community members continue to consult CHWs and take their perspective on their mental health issues<br>Incorporation of new health related behaviors and habits for oneself and family members such as decrease of sugar intake, exercising and breast cancer self-examination<br>Active involvement in one's diagnosis and medical case during medical checkups<br>Increased confidence in discussing health related topics/one's medical condition with the healthcare provider<br>Avoiding delaying the required healthcare visits and medical examinations<br>Increased confidence in managing one's health based on the newly acquired health-related knowledge<br>Providing health-related advices to family members, friends, or acquaintances from the community<br>Prioritizing health in their lives and experiencing encouragement in discussing personal health issues.<br>Seeking practitioner's medical advice instead of referring to non-professionals<br>Engaging in referral of acquaintances in the community to seek medical treatment from healthcare practitioners
6. Individual and Community Empowerment	CHW experiencing self-development and increased self-confidence to seek more career and educational opportunities among CHWs<br>Experiencing empowerment for the ability to support and assist others at the community-level.<br>Increased sense of purpose and responsibility among trained CHWs to offer community members the new information that they have acquired<br>CHW played an active role in engaging with community members and transmitting information to people in their surroundings through formal settings in the COP sessions and afterwards through informal settings<br>CHW served as a role model in her community by engaging in healthy behavior herself and encouraging others to adopt same healthy behaviors<br>CHWs were viewed by community members as acting confidently and without hesitation when addressing the conditions of others<br>Community member's increased awareness on accessing health care facilities that provide low cost or free of charge services<br>Well-equipped to learn and disseminate mental health information.<br>CHWs were observed to be responsive to community's health needs<br>***Subtheme: Psychological improvement and social cohesion***<br>Increase in sensitivity and becoming more accepting towards others<br>Improvement in physical and social functioning<br>Positive progress in family and community processes<br>Feeling better equipped and more in control when it comes to own psychological state<br>Improvements in health and well-being at the personal level<br>Awareness of one's own role related to the psychosocial support process for family members and neighborhood<br>Self-determination and ability to take real decisions<br>Strengthening community and family Support<br>***Subtheme: Career and professional development***<br>Experiencing an empowerment to bring about a real difference as the training content was viewed as significant and applicable to the field of practice<br>Organizing casual meeting for neighborhood members to talk about personal challenges and mental health issues.<br>CHW planning to further benefit others by implementing psychosocial support activities to children in her work of scope and to women in her community who didn't attend the COP sessions<br>CB training motivating learner to pursue a major degree in psychology
7. Significance of CoP Program	COP session perceived to be beneficial for the community as trained CHWs believe that community members are in need of this information<br>Community members were content and satisfied with the topics covered in COP sessions and felt that the topics covered were relevant to the needs of the community

		Capacity building and COP initiative accounting to the needs of vulnerable populations with an emphasis on gender considerations
		The capacity-building training contributed to a change in CHWs perspectives about mental health and allowed them to place it as a priority in their life.
		Improvement of mental health symptoms and functioning at the CHW and community members' level
		Significant improvements of well-being at the individual and community levels
		Strengthening community and family support
		Post COP - Community member experienced a development self-esteem, confidence, and ability to make decisions
		Appreciation for MUH efforts in arranging the awareness activities and targeting women's, elderly's as well as children's health
		Experiencing an enhancement in social connections and relationships with family members, friends and acquaintances
		Subtheme: Factors encouraging access to health services following COP sessions
		Community members receiving awareness on the existence of mental health support services in the community
		Affordable health services are available in the community
		Increased community awareness of health-related problems
		Consistent follow-up by the CHWs increases motivation in community members to access health services
		Community members' trust in CHWs who acquired a certificate from a reputable institution (AUB)

Category	Theme	Code
**Challenges**	1. Learning Modality	Provided Booklets did not contain all the needed information forcing the CHW to refer to other modalities to gain more information
		Need for more engaging and interactive approaches such usage of more videos in order to sustain attention
		Videos mostly presented written information and were majorly used for the theoretical presentation of the content rather than practical.
		A longer duration for the training was needed to be able to cover a wider scope of mental health topics and to provide more practical discussions
		The content was harmonious between the COP and the MUH training, but needed to be more elaborately discussed in the COP sessions
		Some trainees wanted more sessions that target mental health topics related to children as well as men
		More practical and hands-on training could be useful in applying the knowledge learned
	2. Training Logistics	CHW desired more time to deliver content for COP activities, while others thought that the sessions were too long
		Although CHWs appeared to be enthusiastic and eager to provide the content, they occasionally had to be cut due to time restrictions
		CHWs were not always responsive in answering the community members questions during the COP awareness sessions
		Trained CHWs were sometimes nervous to present in front of large number of attendees and thus faced difficulty in sharing the information
		Some CHWs felt that they needed more training and preparedness before leading sessions and engaging in face to face interaction
		There is a need for participants to have easier access to the material they learned after the training to enhance long-term retention
		The schedule of the training was challenging to some attending community members specifically mothers
		Difficulty to reach the location site of the training for some CHWs and community members
		Negative feedback on bringing along kids to the activities
		Physical place was uncomfortable, small and its location was not favored by some of the CHWs
	3. Community Response	Some members were resistant and not receptive to the mental health-related advices to seek professional mental health support provided by the CHWs
		Stigmatized mental health beliefs act as barriers in accessing mental health care facilities or services
		Mothers in the community lack sufficient knowledge about the psychological components of their children's maladaptive behaviors
		Community members' lack of understanding of the role of CHWs in the community restricted their ability to trust the CHWs.
		Inability of a woman to visit a doctor (especially reproductive health specialists like gynecologists) due to husband's refusal and limited awareness exhibited by males
		Women prioritize their children's health ahead of their own, preventing them from getting proper treatment in time.
	4. Limitations to Accessing Health Services	Limited knowledge of the available healthcare centers and services
		Limited availability of psychotherapists and psychological assessments in healthcare/organizations dispensaries across Lebanon.
		Financial barriers to seeking health services
		Limited availability of mental health services in remote areas with limited transportation resources to reach the locations
		Long waiting list and slow follow-up with referred community members aggravates their cases
		Lack of formal affiliation of the CHWs to a specified organization hinders CHW's ability to approach NGOs to follow-up on referrals
